# Dorsal laminectomy for the treatment of lateralised cervical intervertebral disc extrusions in dogs—Prognosis and complications

**DOI:** 10.3389/fvets.2024.1365020

**Published:** 2024-04-22

**Authors:** Diogo Gouveia, Giunio Bruto Cherubini

**Affiliations:** ^1^Dick White Referrals - Linnaeus, Cambridgeshire, United Kingdom; ^2^Department of Veterinary Sciences, Veterinary Teaching Hospital “Mario Modenato, ” University of Pisa, Pisa, Italy

**Keywords:** dorsal laminectomy, cervical, extrusion, prognosis, complications

## Abstract

**Objective:**

Describe the complication rate, expected hospitalization time and prognosis associated with dorsal laminectomy for the treatment of lateralised cervical intervertebral disc extrusion (IVDE) in dogs.

**Methods:**

This is a single-center retrospective case series study. Databases were reviewed from 2012 to 2022 for dogs that had a dorsal laminectomy to treat a lateralised cervical IVDE. Dogs were excluded if additional surgical techniques were performed, or other comorbidities were found on MRI.

**Results:**

Fifty two dogs were included the study. French bulldogs represented 28.8% of the cohort. Patient median age was 6 years and median weight 15 kg. Thirty-five dogs (67.3%) presented clinical signs for <3 days and almost half (44.2%) were ambulatory but presented cervical pain and neurological deficits. Median surgical time was 85 min. Minor intraoperative complications were reported in 22 (42.3%), with hypothermia being the most common. Thirteen (25%) needed revision surgery due to persistent cervical pain with (9/13) or without (4/13) neurological deficits. Re-extrusion or persistent extrusion was found in 92.3% of cases needing surgical revision. Median hospitalization time was 6 days. Forty-seven (90.4%) cases had a good outcome.

**Conclusions and clinical significance:**

Despite the relatively high rate of intraoperative complications and cases needing revision surgery, dorsal laminectomy as surgical treatment for lateralised cervical IVDE is still associated with good long-term prognosis in most of cases. Prognosis is good even when revision surgery is necessary but expected hospitalization time seems to be higher when compared to an alternative surgical technique.

## 1 Introduction

Intervertebral disc extrusion (IVDE) is a common disease of the spinal cord of dogs (1–3).

In dogs, the thoracolumbar spine (T3-L3 spinal cord segments) is the neuroanatomical region most frequently affected by IVDE, the cervical spine can be affected in up to 25% of the cases (2, 4, 5).

Amongst breeds affected by cervical IVDE, Dachshunds, French Bulldogs, Beagles, and Poodles are overrepresented (5–7). Within larger and non-chondrodystrophic breeds, the Labrador and Rottweiler seem more commonly affected (3).

Extrusion (Hansen type I) of the intervertebral discs can lead to compressive myelopathy and clinical signs that can range from cervical hyperesthesia to ataxia, tetraparesis and, in more severe cases, tetraplegia (6, 8, 9). Nerve root signature can also occur with lateralised lesions (10).

Surgical treatment is frequently necessary to relieve pain or improve ambulation and currently there are several described surgical techniques that allow the decompression of the spinal cord in the cervical region (3, 11). Lesion location within the vertebral canal helps elect the surgical technique that is more likely to provide satisfactory access to the extruded material (11).

The ventral slot procedure is the preferred for ventrally located IVDE (12). However, in the case of lateralised cervical extrusions, the ventral slot technique does not seem to provide adequate exposure to achieve effective decompression (13). In such cases, a dorsal laminectomy should be considered (11). A complete dorsal laminectomy or a dorsal hemilaminectomy can be performed during a dorsally based approach to the vertebral canal, with the selection between these techniques being based on the location and amount of space needed to address the target lesion in the vertebral canal (11).

This technique allows the exploration of the vertebral canal and the removal of herniated intervertebral disc material located in the dorsal or lateral aspect of the canal (14).

Previous studies suggest that in addition to being a more difficult and time-consuming procedure, cervical dorsal laminectomy also seems associated with more frequent post-operative worsening of the neurological status (15–17).

The objective of this study was to describe the rate of intraoperative complications, mean surgical and hospitalization time, and outcome associated with dorsal laminectomy performed in dogs with a cervical lateralised IVDE.

## 2 Material and methods

The surgery records from the neurology department at Dick White Referrals were reviewed from January 2012 to July 2022.

Dogs that had a dorsal laminectomy to treat a lateralised cervical IVDE were initially selected.

The exclusion criteria included the absence or incomplete patient records, regarding patient signalment, onset of clinical signs, neurological condition at presentation, intraoperative records and post-operative follow-ups. Patients that required an additional surgical procedure (e.g.: dorsal laminectomy in association with ventral slot and spinal stabilization) or that had possibly clinically significant findings on MRI (e.g.: arachnoid cysts) in addition to the IVDE were also excluded due to the possible impact on postoperative morbidity and outcome. Initial consultation was carried out by different clinicians. Patient anamnesis was obtained before a complete physical and neurological examination was performed.

Neurological grade at presentation was defined based on the 5-Point Scale for spinal cord injury described by Wheeler and Sharp (18). Additional changes to this scale were made to help with case standardization. According to the scale used, grade 0: normal patients; grade 1: patients only experiencing spinal pain; grade 2: patients with ambulatory paresis, any degree of ataxia or lameness, with or without pain; grade 3: patients with non-ambulatory paresis, with or without pain, ataxia and lameness; grade 4: plegic patients with intact deep pain nociception; grade 5: plegic patients with absent deep pain nociception.

After neurological examination the patients had an MRI scan that confirmed the diagnosis of a lateralised IVDE affecting the cervical spinal cord. Only patients with extrusions caudal to C2 and cranial to T1 vertebrae were included in this study.

Decision regarding surgical treatment was based on the presence and severity of neurological deficits (e.g.: non ambulatory tetraparesis or tetraplegia), persistence of clinical signs despite medical treatment (e.g.: cervical hyperesthesia unresponsive to pain relief medication and exercise restriction) and volume of extruded disc material identified on MRI scan.

Dorsal laminectomies were performed after the MRI scan, either during the same general anesthesia or in the following day at a second anesthetic procedure.

A complete dorsal laminectomy was performed after an initial dorsal surgical approach to the vertebral column. A high-speed drill was used to remove the entire dorsal lamina and the interarcuate ligaments were excised using a scalpel blade. The width of the dorsal laminectomy was limited by the articular facets, which were preserved.

In some cases, due to the location and extension of the intervertebral disc material in the vertebral canal, a partial laminectomy (hemilaminectomy) was performed. In these cases, the ipsilateral articular facet was partially or completely removed, to increase the exposure and visualization of the dural tube. The contralateral dorsal lamina and articular facet were preserved.

Surgeries was performed by different surgeons with different degrees of experience, including diploma holders from the European College of the Veterinary Neurology (ECVN) and clinical fellows in Neurology (post-residency trained clinicians).

After surgery patients remained hospitalized until there was confirmation of voluntary urination, low pain scores and evidence of improvement of the neurological status. Between 12 and 24 h after surgery and if pain was adequately controlled, patients would receive one to two physiotherapy sessions during their time in hospital.

Intraoperative complications were collected by reviewing the anesthesia records and documented comments by the anesthetist in charge.

Revision surgery was considered and performed if cervical hyperesthesia persisted, or neurological deterioration was noted after an initial improvement and based on MRI post-surgical features. Revision surgeries were performed by the same surgeon that undertook the initial procedure.

Follow up after discharge was made by re-examination consultation 4 weeks after surgery or, in some cases, through phone call with the owner.

Patient's outcome was classified in 3 groups: (1) Excellent/good outcome was defined as dogs that recovered completely after surgery and presented no abnormalities on neurological examination during revisit consultations or patients that had minor neurological deficits identified in such visits (e.g.: residual monoparesis or ataxia) that did not present an impact in their quality of life; (2) fair outcome was attributed to patients that had persistent neurological deficits on re-examination consultations that caused mild to moderate impact in patients' quality of life (e.g.: moderate ataxia or paresis, leading to a less functional gait); (3) poor outcome was assigned to patients that presented severe neurological deficits after surgery or at re-examination consult and that were not compatible with an acceptable quality of life (e.g.: persistent tetraplegia). Patients that were euthanised due to persistence or deterioration of clinical signs were also assigned to this group.

Statistical analysis was performed using a statistical software package SPSS (Statistical Package for the Social Sciences, v. 26; SPSS Inc, Chicago, Illinois, USA). The normal distribution of the data was assessed analytically, using the Shapiro-Wilk test. For normally distributed data we reported the mean, while non-normally distributed data were presented using medians.

## 3 Results

Seventy one dogs had a cervical dorsal laminectomy performed during the time reviewed. Fifty two dogs met the inclusion criteria.

### 3.1 Patient signalment and characteristics

Dogs of 15 different breeds were included in the study. The French bulldog (*n* = 15), Cocker Spaniel (*n* = 8), and Beagle (*n* = 6) were the most represented breeds and together consisted in 55.7% of the study cohort. Other breeds included the Dalmatian (*n* = 4), Dachshund (*n* = 3), Crossbreed (*n* = 3), Cavapoo (*n* = 2), Cockapoo (*n* = 2), Labrador (*n* = 2), Springer Spaniel (*n* = 2), Cavalier King Charles Spaniel (*n* = 1), Lurcher (*n* = 1), Rottweiler (*n* = 1), Shih Tzu (*n* = 1), and Weimaraner (*n* = 1).

Patients had ages between 2.9 and 11.9 years with a median of 6 years. The median body weight of the patients was 15 kg, with a minimum of 3.9 kg and a maximum of 42 kg recorded. Most of the dogs (*n* = 44) had an ideal (4 or 5) or just slightly above ideal (6) body condition score (BCS). Overweight dogs (BCS > 6) represented 13.5% (*n* = 7) of our cohort and only one patient was underweight (BCS of 3/9).

Male dogs constituted 59.6% (*n* = 31) and females were represented in 40.4% (*n* = 21) of the cohort. Most patients were neutered (*n* = 38, 73.1%), with only 26.9% (*n* = 14) being entire.

### 3.2 Clinical presentation and affected IVD space

Clinical signs were noted with a median of 3 days previously to the referral consultation, with most dogs being managed by the referring vets during that period.

The great majority of dogs were seen for referral after clinical signs were noted for 3 days or less (*n* = 35, 67.3%). Twelve (23%) were referred after presenting signs of possible cervical myelopathy for more than 3 days and up to 2 weeks. In three dogs (5.8%), clinical signs were present for 2 to 4 weeks. The two patients with the longest period of clinical signs before presentation showed evidence of cervical hyperesthesia 45 and 60 days before presentation.

Grade 2 spinal cord injury was the most commonly identified (*n* = 23) and only 14/52 patients were non ambulatory. Grade 1 spinal cord injury was reported in 15/52 (28.8%) and grade 3 was seen in 13/52 (25%). Only one patient presented in grade 4 and there were no reported grade 5 lesions (Table 1).

**Table 1:** Grade of spinal cord injury.

	**Frequency**	**Percentage**
Grade 0	0	0%
Grade 1	15	28.8%
Grade 2	23	44.2%
Grade 3	13	25%
Grade 4	1	1.9%
Grade 5	0	0%

The C4-C5 (*n* = 18), C5-C6 (*n* = 14) and C3-C4 (*n* = 10) accounted for 79.2% of all the IVDE reported in this study. The C2-C3 (*n* = 6), C6-C7 (*n* = 4), and C7-T1 (*n* = 1) IVD spaces were affected less often. There was a similar distribution on the lateralisation of the lesions between the left (51.9%) and right (48.1%) side and IVDE affecting more than one IVD space was reported in only one patient (C4-C5 in combination with C5-C6), leading to a total of 53 IVDEs between the 52 patients.

### 3.3 Surgical time and intraoperative complications

Surgical time was widely variable. The quickest surgery took 40 min, while the longest took 190 min to complete. If divided in specific groups, 19.2% (*n* = 10) were completed in 60 min or less; 38.5% (*n* = 20) took between 60–90 min; 21.2% (*n* = 11) were performed in 90–120 min and another 21.2% (*n* = 11) lasted more than 120 min.

The median surgical time was 85 min, increasing to 100 min (70–190) if only dogs with a body weight superior to 15 kg (*n* = 25) were assessed.

Surgeries were performed by 8 different surgeons.

The dorsal laminectomy more frequently involved two adjacent vertebrae (*n* = 48). Less commonly, the extension of the IVDE indicated a wider approach [involving three (*n* = 3) or four (*n* = 1) vertebrae]. Forty-five patients received a complete dorsal laminectomy, while seven had a dorsal hemilaminectomy.

Intraoperative complications were reported in 42.3% (*n* = 22) cases and these included mainly hypothermia (*n* = 14), hypotension (*n* = 8), and significant hemorrhage (*n* = 5). Regurgitation (*n* = 3), hypoventilation (*n* = 3), and bradycardia (*n* = 1) were rarely reported. 57.7% (*n* = 30) cases had no reported complications during surgery.

Fenestration was not performed during the initial surgery, due to associated technical difficulties.

### 3.4 Revision surgery

One quarter of the patients (*n* = 13) needed to go through a revision surgery, due to persistent of pain (*n* = 4) or persistence of pain and absence of neurological improvement or neurological deterioration (*n* = 8). One case needed revision surgery due to onset of dyspnoea during the recovering period.

Of the 13 revised cases, 4 had a ventral slot and spinal stabilization during the second surgery and 1 had fenestration of the affected IVD space during the dorsal approach. The remaining 8 only had simple revision of the dorsal laminectomy surgery with no additional procedures performed.

**Figure 1:**
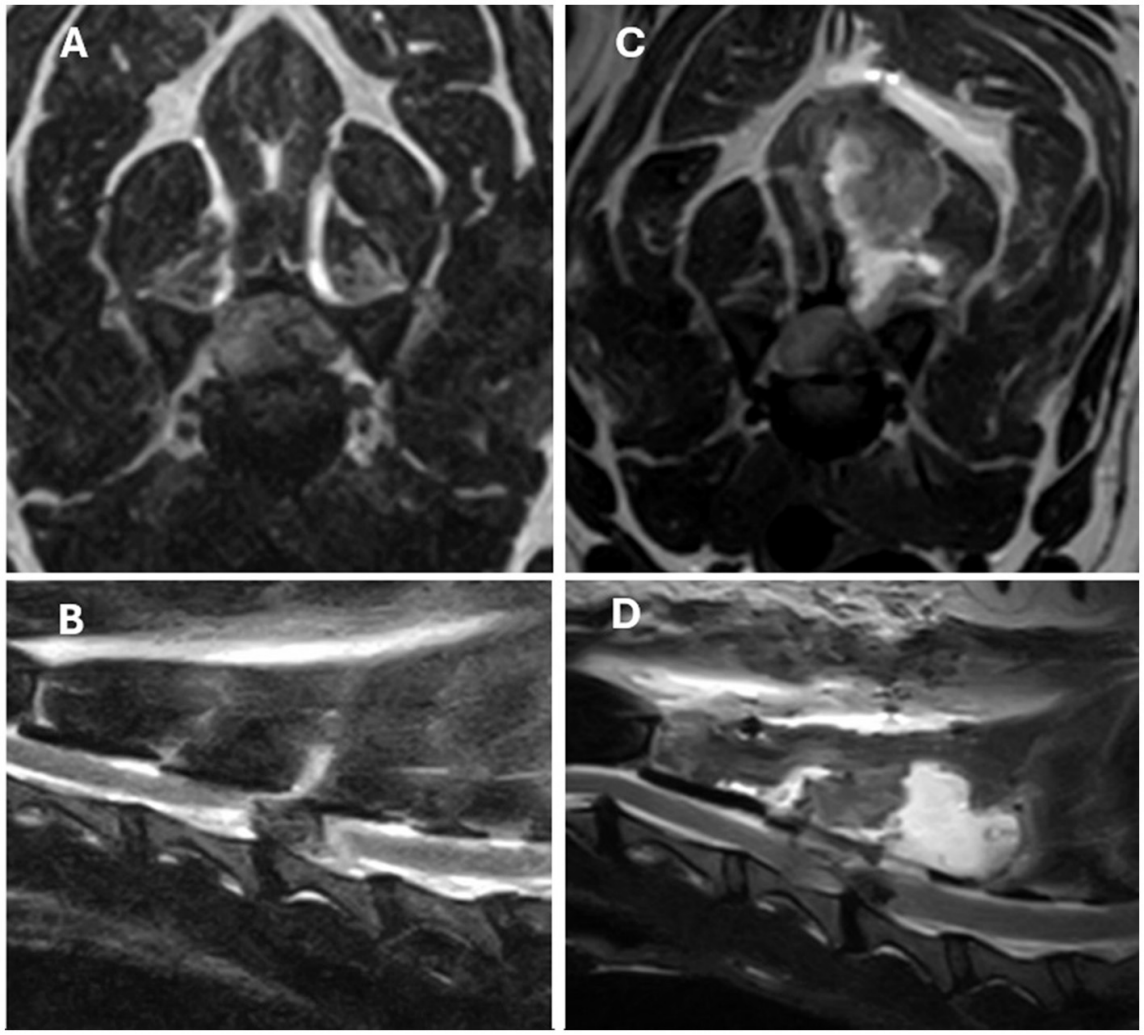
T2-weight MRI images of a left lateralised C4-C5 IVDE in a 9-year-old Beagle. **(A, B)** Are pre-operative transverse and sagittal images. **(C, D)** Are post-operative transverse and sagittal images obtained 3 days after surgery due to worsening of the neurological status. Left, ventrolateral extradural spinal cord compression secondary to suspected re-extrusion at the previous surgical site can be identified in images **(C, D)**. Extensive muscular and subcutaneous changes consistent with seroma formation and/or hemorrhage can also be seen but were considered likely to be not clinically significant.

**Figure 2:**
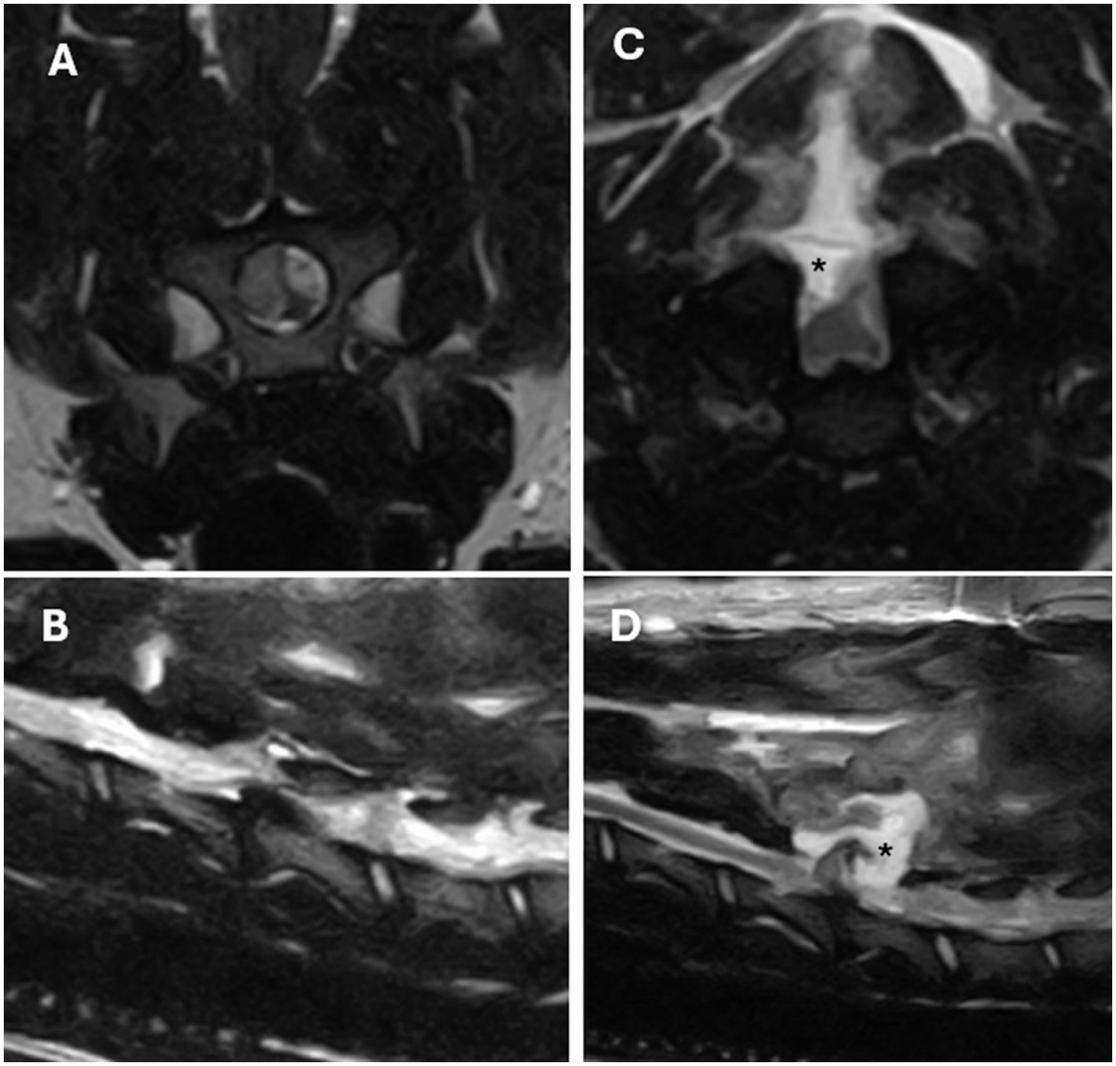
T2-weight MRI images of a left lateralised C5-C6 IVDE in a 4-year-old Dalmatian. **(A, B)** Are pre-operative transverse and left parasagittal images. **(C, D)** Are post-operative transverse and sagittal images obtained 3 days after surgery due to worsening of the neurological status. Post-operative changes suggestive of seroma formation (*) causing secondary dorsal compression of the spinal cord can be identified in **(C, D)** images.

Re-extrusion or persistent extrusion (Figure 1) was identified in 92.3% (*n* = 12) of cases and of these, 41.6% (*n* = 5) had a significant degree of seroma (Figure 2) and/or haematoma in combination with the herniated disc material. A large compressive haematoma without associated extruded disc material was identified in only one case.

### 3.5 Hospitalization time

After surgery, patients spent a median of 6 days in the hospital, with a minimum of 2 days and a maximum of 14 days. If patients that needed revision surgery are excluded, the median hospitalization time drops to 4 days. A maximum stay of 12 days was recorded for this group of patients. On the other hand, the median hospitalization time for patients that required revision surgery was of 8 days, with a minimum stay of 3 days and a maximum stay of 14 days.

The median time for revision surgery to be performed was of 3 days.

### 3.6 Outcome

**Figure 3:**
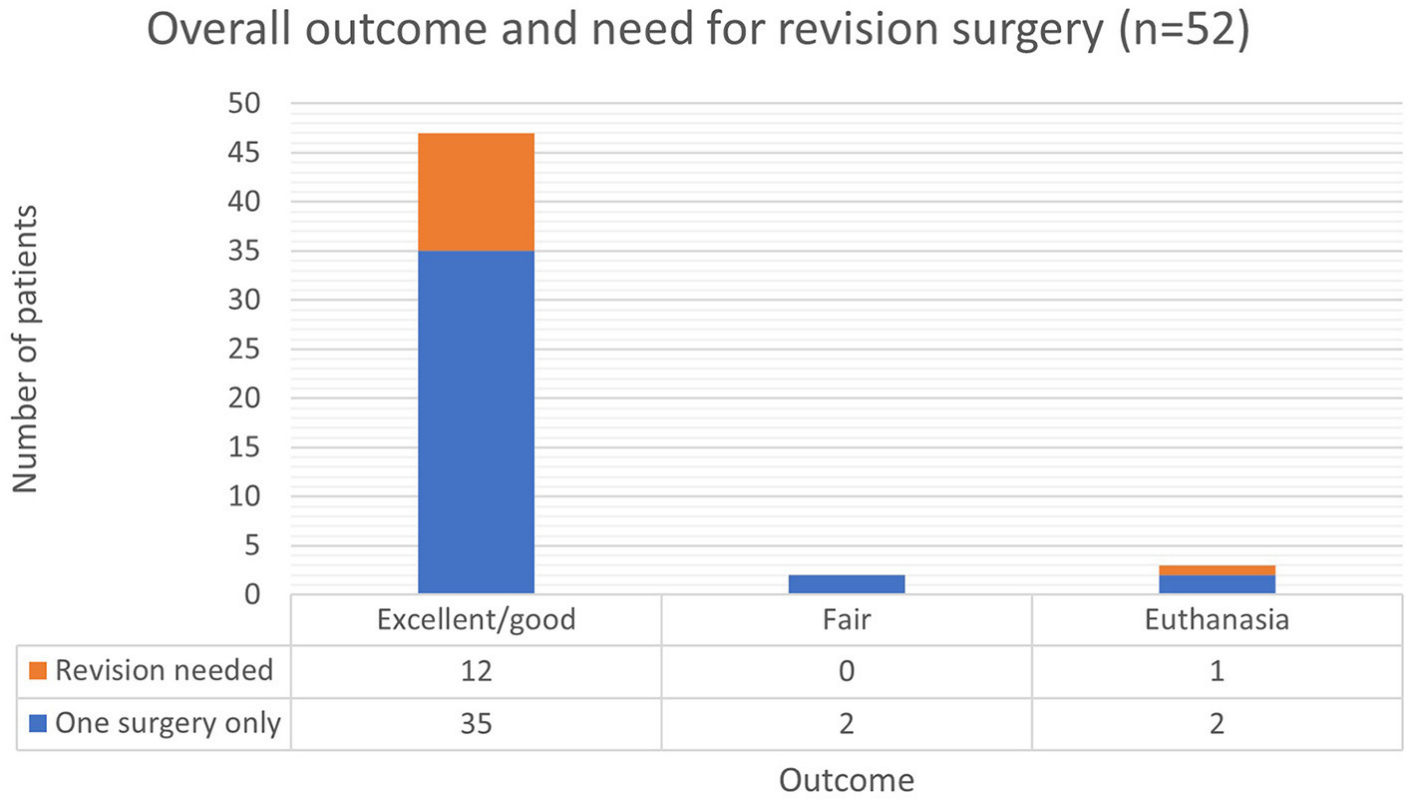
Outcome and need for revision surgery.

Most patients (*n* = 47, 90.4%) that required a cervical dorsal laminectomy had an excellent/good outcome (Figure 3). Regardless of their initial grade of spinal cord injury, on follow-up consultations 1 month after surgery these patients would be independently ambulatory. On examination they revealed no neurological deficits or only residual ataxia or paresis that would not impact their ambulation or quality of life.

Two dogs had a fair outcome and continue to present a significant degree of paresis at the last follow-up about 8 weeks after surgery. This had a moderate impact on patients' ambulation and quality of life.

Almost all patients (12/13, 92.3%) that required a revision surgery ended up having an excellent/good outcome in the long term.

Three patients were euthanized due to persistence or deterioration of clinical signs. One was euthanized after revision surgery due to persistent hypoventilation.

From the two other patients that were euthanized after the first surgery (Figure 1), one of these presented with grade 1 of spinal cord injury and become tetraplegic, with pyrexia and melena also developing after surgery. The other euthanized patient presented with grade 3 of spinal cord injury and progressed to grade 4 with hypoventilation after surgery. Revision surgery was not attempted in any of these cases.

## 4 Discussion

Currently, studies describing the complication rate and outcome associated with a complete dorsal laminectomy or hemilaminectomy approach to lateralised cervical intervertebral disc extrusions are very limited (19–21).

Different techniques are currently described to provide dorsal decompression of the cervical spine. These involve complete (dorsal laminectomy) or partial (dorsal hemilaminectomy) removal of the dorsal lamina. The articular facets might also be partially or completely removed, if necessary, although bilateral facetectomy tends to be avoided due to the resultant and undesirable degree of vertebral instability created (11).

While various lateral approaches to the cervical spine have been previously outlined, these are generally regarded as technically more challenging (22).

More recently, Santifort et al. (23) described a modified unilateral dorsal laminectomy technique that can constitute an alternative to the techniques previously described. This approach involves the removal of the dorsal lamina of one or more cervical vertebrae, with the laminectomy being limited medially by the spinous process or midline raphe and laterally by the caudal articular process. Some of the considered advantages of this approach include a decreased risk of vertebral artery and spinal cord injury (23).

A dorsal approach to the cervical spine can be considered to address dorsally or laterally located lesions within the cervical vertebral canal (14, 15, 20), since the ventral slot technique does not allow a good access to intervertebral disc material located in these areas of the vertebral canal (13).

When compared to ventral slot decompression, cervical dorsal laminectomies are historically considered to be a more invasive surgical technique, associated with more complications and increased hospitalization time (15–17). Early postoperative deterioration was also previously reported (15–17).

### 4.1 Patient signalment and characteristics

About 16–25% of all canine intervertebral disc extrusions occur at a cervical level with Dachshunds, Beagles and Poodles being frequently overrepresented (5).

Age distribution of our cohort was in accordance with that reported in current literature, with all dogs being older than 2 years old (3). The mean body weight reflects the higher prevalence of medium sized breeds (e.g., French bulldog, Cocker Spaniel, and Beagle).

Most of the dogs in our cohort had an ideal or only mildly increased BCS, suggesting that IVDE can occur frequently in patients with adequate body condition.

Male dogs were only marginally more prevalent possibly reflecting the known lack of strong sex predilection in cases of IVDE (3).

### 4.2 Clinical presentation and affected IVD space

The rate of onset of IVDE is expected to influence the severity of the myelopathy due to the increased contusive injury in higher velocity disc extrusions (24). Slower rates of onset of IVDE were suspected to be related with the slower development of signs of cervical myelopathy seen in our study. Similarly, an initial more conservative approach done by the referring veterinarian was also expected to influence the gap between onset of clinical signs and referral consultation seen in a small fraction of the dogs in our cohort.

Similarly to a study by Schmied et al. (25), the dogs in our cohort were more frequently ambulatory tetraparetic and/or with some degree of forelimb lameness. This is suspected to be due to the lateralised nature of the disc extrusions in these cases, which seems to less commonly lead to severe spinal cord compression to cause non ambulatory tetraparesis or tetraplegia.

### 4.3 Surgical time and intraoperative complications

A study by Rossmeisl et al. (26) including 546 dogs going through cervical ventral slot technique due to intervertebral disc extrusion identified adverse effects in only 9.9% of the cases. Of these, only 6.4% were major complications (e.g., deterioration of the neurological status, significant intraoperative hemorrhage, or persistent pain).

Intraoperative complications were relatively frequent in the patients of our study but most of these were minor and easily controlled during surgery. Major or life-threatening intraoperative complication were not reported in any patient.

Our study included different surgeons with different levels of experience, and this was expected to impact surgical time significantly. Similarly, there was also a very meaningful variation in patient size which will impact the extent of soft tissue dissection needed to approach the cervical vertebrae, influencing not only the surgical time but also some of the possible intra and postoperative complications (e.g., intraoperative hemorrhage, haematoma, or seroma formation).

Although major intraoperative complications were not reported in any case, significant iatrogenic spinal cord injury could be suspected in the three euthanized dogs, due to the severe neurological deterioration after surgery (progression from grade 1 or 3 spinal cord injury to grade 4). Due to the low number of cases that did not improve completely or were subjected to euthanasia a correlation between breed/size or BCS and a possible poorer outcome could not be established. A predisposing factor for possible increased intraoperative iatrogenic trauma could also not be determined.

### 4.4 Revision surgery

The number of patients requiring revision surgery was significantly increased when compared to other surgical techniques, with only 3.1% of dogs that received a ventral slot technique needing revision surgery, mainly due to surgical technical errors (26).

Almost all patients (12/13) undergoing revision surgery had more extruded disc material removed from the vertebral canal at the time of revision. It was not possible to clarify if this was related with persistent disc material not completely removed in the first surgery or related with a re-extrusion process.

Unsuccessful spinal decompression after the first surgery is also a possible cause for the need of revision surgery in some of our patients. Extensive intraoperative bleeding could make the visualization of disc material challenging and limit exploration of vertebral canal. Although this was not reported as a recurrent complication in the anesthesia records, intraoperative hemorrhage significant enough to impact the surgeon's operative view might still have occurred and been overlooked in our patients.

One patient required revision surgery 41 days after the dorsal laminectomy and initial improvement of his neurological condition. This was suspected to be due to a late re-extrusion process. The need for early revision surgery in the remaining (12/13) patients due to deterioration or lack of improvement of their neurological condition provides further evidence for the failure of the initial surgical procedure in adequately achieving decompression of the nervous structures, preventing early re-extrusion, or for contributing to significant iatrogenic trauma to the central nervous system.

Aikawa et al. (27) suggested that at the level of the thoracolumbar spine, prophylactic fenestration seems to help decreasing the risk of future herniation in the treated intervertebral discs, also reducing the likelihood of need for a second surgery. Despite this, a systematic review by Pontikaki et al. (28), failed to reach a conclusion on the value of prophylactic fenestration on the recurrence of dogs treated surgically for a thoracolumbar IVDE. The low quality of the available evidence on the topic was regarded as the cause for this question to remain unanswered.

Cervical spinal decompression via ventral slot technique seems to virtually eliminate the risk of recurrence of IVDE at the operated intervertebral disc space (29) due the removal of most of the nucleus pulposus during this surgical approach (30). For this reason, recurrence in these patients tends to almost exclusively happen in adjacent IVD spaces (29).

Studies evaluating the efficacy of prophylactic fenestration of cervical intervertebral discs in preventing recurrence of disc herniation are currently lacking (29, 31). Nonetheless, the ventral approach to the cervical vertebral column allows the surgeon to possibly fenestrate adjacent intervertebral disc spaces, if desirable. This is not easily performed with a dorsal approach to the vertebral canal, as done during a dorsal laminectomy. Extensive and undesirable spinal cord manipulation would be necessary to gain enough visualization of the dorsal aspect of the anulus fibrosus through the vertebral canal and perform fenestration. The efficacy and safety of this fenestration technique was never evaluated and the possible complications associated with excessive spinal cord manipulation seem to preclude the realization of such study.

The lack of fenestration of the affected IVD space in our patients might have predisposed them to early re-extrusion and lead to the need for revision surgery in a significant proportion of the cases and this might constitute another disadvantage of this technique.

Performing a second ventral approach to the cervical spine to allow fenestration, before or after decompressive dorsal laminectomy should be considered, but it is important to take into account the increase in surgical time in the absence of strong evidence to support the prophylactic effect of this procedure in preventing the recurrence of IVDE in the cervical spine.

### 4.5 Hospitalization time and postoperative deterioration

After surgery, the patients in our study spent a median of 6 days in the hospital. This was in accordance with the results previously reported by Taylor-Brown et al. (17) in dogs undergoing a dorsal laminectomy procedure due to IVDE, but seemingly longer than the median of 4 days reported by Rossmeisl et al. (26) after ventral slot surgery.

Taylor-Brown et al. (17) reported neurological deterioration 48 h post operatively in 55% of the dogs that underwent a cervical dorsal laminectomy for treatment of compressive cervical lesions. This study involved several other conditions (neoplasia, vertebral arch anomalies, spinal arachnoid diverticulum, and cervical spondylomyelopathy) and postoperative deterioration was less frequent (22% of cases) when only assessing patients with acute IVDE.

Regrettably, in our study serial assessment of the neurological status of the patients after surgery was not available for review.

Post-operative deterioration could have contributed to an increased hospitalization time in our patients. We believe that the review of the postoperative neurological status of the patients of our study would have been important to better understand the possible causes behind the increased hospitalization time and provide owners of future patients with more accurate expectations in the immediate postoperative period.

### 4.6 Outcome

Some studies report cervical dorsal laminectomy and hemilaminectomy (with removal of articular facets in addition to the interarcuate ligament and dorsal lamina) to be associated with a good outcome in small breed dogs suffering from single or multiple cervical intervertebral disc extrusions (19–21).

Other compressive cervical myelopathies were also effectively addressed with this surgical technique (15, 32).

Our study shows further evidence that dorsal laminectomy seems to be associated with a good to excellent outcome in most dogs, independently of their breed and size.

The need for revision surgery does not seem to impact the overall outcome, with almost all dogs requiring a second surgical approach ending up achieving complete recovery.

Hypoventilation was the leading cause (2/3) for euthanasia in our patients. Hypoventilation was an uncommon complication in our study, supporting the results obtained by Taylor-Brown et al. (17), where only 1 of the 70 dogs receiving a dorsal laminectomy required post-operative mechanical ventilation due to respiratory compromise.

Despite this, hypoventilation still seems to be more common with a dorsal laminectomy when compared to a ventral slot technique. In the study by Rossmeisl et al. (26), only 2 of the 546 dogs that underwent ventral slot decompressive surgery due to a cervical IVDE required postoperative mechanical ventilation due to neurogenic hypoventilation, suggesting this is a rare complication with this technique.

Evidence of hypoventilation in the post-operative period is more commonly observed in patients with severe cervical spinal cord dysfunction (33).

Since hypoventilation was not present in our patients in the preoperative period, the development, and persistence of this complication after surgery could suggest that further spinal cord dysfunction occurred during surgery.

According to the owners' wishes, two of the three euthanized dogs did not undergo a revision MRI scan or any additional surgical procedure. Therefore, it was not possible to clarify the cause behind their neurological deterioration, determine the possible indications for revision surgery, and evaluate the potential impact of this procedure on their outcome.

### 4.7 Study limitations

Our study has several limitations. As a single-centered study, our work reflects the reality of the use of this technique in our hospital but extrapolation to the general dog population should be done with care.

The lack of uniformization of our cohort was also expected to affect some of our variables. This is particularly expected in the surgical time, since dogs of larger size are anticipated to be associated with a more labored surgical approach due to the denser cervical musculature requiring dissection (as suggested in our results). Questions can also be raised as to whether larger breeds might also be associated with increased intra or postoperative complications, when compared to smaller dogs. This might also secondarily impact hospitalization time, with meaningful financial implications to owners.

Detailed serial neurological examinations were not recorded in our patients and for this reason recognition of postoperative neurological deterioration was not possible. Similarly, the degree of postoperative pain and strength of analgesic protocol required was also not evaluated. These variables were considered clinically significant when attempting to characterize a surgical technique and their assessment would have added meaningful value to this study.

The lack of fenestration of the affected IVD space during the initial surgery also made us unable to clarify if this additional procedure could have contributed to a reduced number of cases needing revision, as previous studies have suggested. The surgeries documented in our study were performed by different surgeons, with very variable degrees of experience. Surgical time and intraoperative complications were expected to be influenced by this.

Finally, the low number of dogs with a poor outcome did not allow us to identify possible predisposing factors associated with this. Larger, multicentre studies are required to investigate the existence of a relationship between breed, body weight, BCS or surgeon's experience and the prognosis of dogs with a lateralised cervical IVDE that are treated with a dorsal laminectomy.

### 4.8 Conclusion

Our study provided further evidence that a dorsal laminectomy can be used successfully to approach lateralised cervical intervertebral disc extrusions in dogs. Intraoperative complications seem to be common but mild and the long-term prognosis seems to be good.

Severe intra or postoperative complications might be less frequent than previously thought, but the hospitalization time and proportion of dogs requiring a revision surgery appears to be significantly higher than that expected with a ventral slot technique. Further studies are needed to better understand the reason behind the reported increased hospitalization time, the need for revision surgery, value of concomitant fenestration and assess a possible correlation between patient's signalment and short- and long-term prognosis.

## Data availability statement

The raw data supporting the conclusions of this article will be made available by the authors, without undue reservation.

## Ethics statement

Ethical approval was not required for the studies involving animals in accordance with the local legislation and institutional requirements because this is a retrospective study utilizing electronic clinical records under adherence to relevant GDPR policy with patients anonymized. Formal ethical approval was therefore not required. Written informed consent was obtained from the owners for the participation of their animals in this study.

## Author contributions

DG: Conceptualization, Formal analysis, Investigation, Methodology, Writing – original draft, Writing – review & editing. GC: Supervision, Writing – original draft, Writing – review & editing.
